# Interpersonal Communication Style and Personal and Professional Growth among Saudi Arabian Employees

**DOI:** 10.3390/ijerph20020910

**Published:** 2023-01-04

**Authors:** Lowai G. Abed, Mohaned G. Abed, Todd K. Shackelford

**Affiliations:** 1 University of Jeddah, College of Communication and Media, Department of Communication and Public Relations, Jeddah 23218, Saudi Arabia; 2 Department of Special Education, Faculty of Educational Graduate Studies, King Abdulaziz University, Jeddah 21589, Saudi Arabia; 3 Department of Psychology, Oakland University, Rochester, MI 48306, USA

**Keywords:** interpersonal communication style, personal growth, professional growth, one-way communication, two-way communication, Saudi Arabia

## Abstract

This study explored the association of interpersonal communication style (ICS) with personal and professional growth among Saudi Arabians in the context of the work environment. It was hypothesized that different ICSs are differently associated with personal and professional growth. The participants were 143 Saudi Arabian adults, including members of both genders, who were employed, with varying incomes, and who had different education levels. The participants completed a self-report survey, assessing their ICS and their personal and professional growth in an employment context. Consistent with the hypothesis, the results indicated that different ICSs are differently associated with personal and professional growth. The controlling ICS had the strongest relationship to professional and personal growth, whereas the dynamic ICS had the weakest relationship to professional and personal growth. In the Discussion, we address the limitations of the study and identify several directions for future research, with specific reference to the Saudi Arabian cultural context.

## 1. Introduction

Interpersonal communication refers to the transmission of information between at least two individuals. Interpersonal communication style (ICS) is important for forming and maintaining relationships, whether in a work setting or a private space [1]. A person’s interpretation of messages is based on the verbal and non-verbal codes expressed in the sender’s and the receiver’s communications [2]. These interpretations are based, in part, on the cultural context [3]: for instance, certain facial expressions are interpreted as automatic responses to specific stimuli, whereas other non-verbal cues, such as direct eye contact, may mean something different in different cultures [3]. For these reasons, ICS is important for relationship functioning—which means knowing when and how to communicate, as well as when to express certain non-verbal cues, to send specific messages. The ICSs considered in the current study—controlling, egalitarian, structuring, dynamic, relinquishing, and withdrawal—can evoke certain interpretations by receivers of messages [4].

In work or personal contexts, a particular ICS may help individuals grow or may cause them to regress, in the context of a relationship [5]. ICSs can be categorized as either one-way or two-way communication styles, each associated with benefits and detriments [5]. For instance, one-way communication may hinder personal and professional growth, because it lacks an interactional component [6], whereas two-way or interactive communication allows for both the sender and the receiver to ask questions and clarify issues [6].

### 1.1. Objectives of the Current Study

A key objective of the current study was to identify associations between ICSs and personal and professional growth. The study explored the views that people have regarding various ICSs and how these styles may affect their relationships. Few studies have considered the associations of ICSs with reported psychological and emotional wellbeing in a non-Western sample, generally, and specifically in a Saudi Arabian sample. Thus, this study aimed to contribute to the empirical literature, by investigating the associations between ICSs and personal and professional growth in a sample of Saudi Arabian adults.

### 1.2. Interpersonal Communication Style

Interpersonal communication style (ICS) refers to a “typical set of behaviors an individual displays in their interaction with others” [7] (p. 539). “Interpersonal” refers to relationships and communication, and to the processes therein that shape social interactions. Because interpersonal communication relates to people, individual difference traits are one set of components that may affect interactions with others. Bateman and Zeithaml (1990, cited in [7]) identified six ICSs in a work environment: controlling; egalitarian; structuring; dynamic; relinquishing; and withdrawal. Table 1 presents brief descriptions of these styles.

According to Dwyer and Hopwood [7], managers in work contexts employ a particular ICS based on their personality traits, leadership style, and employment experience. Successful managers may adjust their ICS to meet the needs of the situation or to suit the context. Adaptability, in this case, suggests being open to new opinions or changes in an organization, as well as managing unexpected demands [7].

Interpersonal communication also entails sending and receiving both verbal and non-verbal signals. Ansari [4] argues that interpersonal communication skills (verbal and non-verbal) are important for maintaining interpersonal relationships, and for effective functioning in a work setting. On the other hand, Taylor [8] notes that much of modern communication, particularly organizational communication, is neither face-to-face nor limited to two people, when one considers digitized communication such as emails and video conferencing. The absence of or limitations associated with traditional signals of interpersonal communication can lead to conflict in work settings [9]. These conflicts may be due to misunderstandings or disagreements between managers and employees, or between employees [9]. Workplace conflict and misunderstanding, especially between management and employees, can lead to increased employee turnover [10]. Some of these workplace problems can be traced to poor or limited interpersonal communication by managers [4]. 

Bucata and Rizescu [11] argue that communication management is a domain of leadership that should be attended to by managers, because effective communication management facilitates employee training, harmonization, control, and assessment. The same scholars note that managerial communication plays a significant interpersonal role, because managers serve as leaders while engaging with peers, subordinates, and customers within and outside the organization. Bucata and Rizescu report research indicating that managers use 45% of their time to communicate with equals and employees, and only 10% to communicate with superiors. In addition, successful managerial communication facilitates employee job satisfaction, by enabling sharing of information between managers and employees, as well as enhanced employee performance, by effective communication of organizational objectives [11]. 

### 1.3. One-Way Communication

Communication theory highlights three approaches by which to conceptualize the communication process: one-way (linear); two-way (interactive); or transactional [12]. In each approach, the sender attempts to produce an attitudinal change in the receiver [12]. Feedback is important for many reasons, such as to signal reception of the message and to ensure that the communication has the intended effect [13]. Including feedback in a one-way or linear approach facilitates the effectiveness of the communication [12]. Moreover, it is important to identify whether the communication is formal or informal, even in an organizational context. Awad and Alhashemi [5] found that the formal context is likely to embody a one-way communication channel, especially if the communication is occurring through an official path, such as email, meeting, or text: this suggests a working arrangement that may limit the interactive approach, especially between managers and employees, because of a strict hierarchy within the organization [14]. In addition, there may be an emphasis on written communication that requires answers to specific queries, and does not welcome engaging with other issues [15]. This leads to an “ask-and-obey” approach to communication between managers and employers [15].

### 1.4. Two-Way Communication

In two-way communication, interaction is crucial; nonetheless, there are diverse interpretations of what “interaction” means [12]. Interaction typically refers to a reciprocal dialogue that includes opportunities for individuals to influence each other. This type of communication typically occurs between individuals who are cordial, but may be initiated and pursued by workplace managers who are democratic and transformational [16]. Awad and Alhashemi [5] provided evidence that informal communication in the workplace tends to be two-way, and is not usually sanctioned or regimented in the manner of one-way (especially manager–employee) communication. The authors further noted that informal communication may facilitate positive relationships that serve as a source of influence [5]. However, such informal interactions also can be a source of communication problems, such as when people engage in rumor-spreading [17]. 

Communication is essential to the development and maintenance of interpersonal relationships. A two-way communication approach provides the sender and receiver with opportunities to interact and communicate effectively, by sharing ideas, solving problems, and expressing thoughts and feelings. This communication can be verbal or non-verbal, either of which may include signals of intent [5]. Two-way communication may facilitate better working relationships and reduce misunderstandings that interfere with the personal and professional growth of employees. Ruler [12] argues that two-way communication is essential for effective interaction, as communication is a process of constructing meaning. 

### 1.5. Personal and Professional Growth

Personal and professional growth can be affected by communication, whether in a work setting or in a private space [18]. The style of interpersonal communication that individuals use can affect whether they grow personally and professionally [1]. For instance, a controlling ICS can obstruct personal and professional growth, whereas an egalitarian ICS can support personal and professional growth [19]. Dwyer and Hopwood [7] argue that positive communication is adaptable, in that it expresses that an individual has “an open mind, responds with a positive attitude to change, and is willing to learn new ways to achieve targets and objectives” (p. 540). Based on this argument, individuals may grow personally and professionally when the communication is interactive and thereby provides opportunities to ask questions and to learn. The authors add that skilled communicators can switch ICSs, based on the situation and the people with whom they are interacting [7].

Bucata and Rizescu [11] found that communication from management is typically intended to inform employees, and to guide them towards high-quality workplace performance. Therefore, to communicate well means not only arranging one’s thoughts, but also expressing them in a manner that captures the attention of the receiver [11]. Ansari [4] supports this argument with the contention that effective interpersonal communication skills maintain a healthy and appropriate balance in interpersonal relationships. Previous research (see [4]) notes that effective interpersonal communication skills in organizations result in employee dedication, improved performance, and meaningful work relationships, as well as greater trust among and between employees and management. 

Beenen and colleagues’ [20] study of managerial interpersonal communication skills found that the most effective interpersonal communication is represented in a threefold approach: supporting, motivating, and managing conflict. In addition, managerial interpersonal communication skills predicted attitudes and performance among employees and managers over and above personality traits and leadership style [20]. Thus, managerial ICS can affect how employees spend their time in the workplace, and whether they experience joy or despair, perform well or poorly, or are healthy or ill [20].

### 1.6. Conceptual Framework

Interpersonal Need Gratification theory [21] addresses relationships at work, and how they influence personal and professional growth. The theory posits that psychological needs are arranged in a hierarchy, and can affect the associations between job factors and satisfaction [21]. Thus, individuals enter a relationship based on what they seek to gain. For instance, an employee can form a relationship with a manager to find favor, or to learn and grow in the work context. Awad and Alhashemi [5] studied the effects of interpersonal communication on employee dedication, and found that when employee needs are met through satisfying communication, they are likely to develop successful relationships, and to experience satisfaction in their work. The authors also noted that Interpersonal Need Gratification theory is goal-oriented insofar as people need inclusion, control, and affection [5]. This may explain why personal and professional growth can depend on the type of relationship individuals have with others—which influences ICS. 

### 1.7. Hypothesis

Based on Interpersonal Need Gratification theory, individuals may sometimes attempt to endear themselves to workplace superiors, to gain favor. Thus, employees may be more likely to listen to and yield to their superiors. Furthermore, yielding to superiors provides an opportunity for employees to learn and grow, as their superiors are usually more experienced in the workplace. Therefore, some ICSs may have a stronger influence on employees than others, based on what employees hope to achieve—that is, whether to find favor or to learn and grow. We offer the following hypothesis, which we investigated in a sample of Saudi Arabian adults: different ICSs are differently associated with personal and professional growth.

## 2. Methods

### 2.1. Study Design

A correlation design was used to investigate relationships among the target variables: in addition, an ANOVA single-factor analysis approach was employed to investigate these relationships. The independent variables included the different ICSs and demographic variables, and the dependent variable included an assessment of personal and professional growth. We tested the following hypothesis: different ICSs are differently associated with personal and professional growth.

A Likert-scale self-report survey (described below) was used to collect the data. The items considered by the current study had 5 ordinal response options: “strongly agree”; “agree”; “uncertain”; “disagree”; and “strongly disagree”, coded 5 to 1, respectively. ANOVA single-factor analysis was applied, to measure the variances associated with the ordered levels. 

### 2.2. Participation and Procedure

The survey was administered by convenience sampling of 192 adult members of the public in Saudi Arabia. The survey included several demographic questions. More women than men participated, with the greatest number of participants in the age group 31–50 years. Exploratory demographic questions assessed level of education and monthly income. Table 2 presents a summary of available demographic information for the convenience sample of Saudi Arabian adults.

The participants were issued a survey after the researcher described the purpose of the study, and following provision of informed consent. Participation was voluntary and was not rewarded. The participants were instructed to answer items by selecting the response option that best reflected their answer. Although the survey was distributed to 192 participants, 49 participants responded to the first question but then discontinued participation: the final sample that provided complete data was thus 143.

Convenience sampling guided the pseudo-random sampling of participants in public spaces, with some subjective bias unavoidable in our efforts to select adults 18 years of age or older, who appeared likely to be employed. Following Dwyer and Hopwood [7], the data were coded to differentiate six ICSs: controlling (CS); egalitarian (ES); structuring (SS); dynamic (DS); relinquishing (RS); and withdrawal (WS). Each ICS was indexed by the item identified in Table 3.

### 2.3. Ethical Considerations

The study was conducted in accordance with the Declaration of Helsinki, and approved by the Research Ethics Committee of King Abdulaziz University (protocol KEP-92-120-42; date of approval: 15 February 2021). Written informed consent was obtained from those participating, prior to their participation. Due to the nature of this research, the participants did not agree for their identified data to be shared publicly. The authors declare no conflict of interest. The funders had no role in the design of the study, in the collection, analyses, or interpretation of the data, in the writing of the manuscript, or in the decision to publish the results.

## 3. Results

Table 4 presents the descriptive statistics for personal and professional growth by ICS. For analytic and reportorial efficiency, “level” differentiates the strength of the reported personal and professional growth, assigned according to average growth. These findings indicate that, for example, the respondents reported high growth professionally and personally when directed by experienced individuals (CS). As another example, respondents reported moderate growth professionally and personally when encouraged to take action by their superiors (DS). 

The ANOVA results (see Table 5) showed a *p*-value of 0.04624, which was evidence for rejecting the null hypothesis, as the value was less than 0.05. This suggests that reported personal and professional growth differs with ICS. 

## 4. Discussion

The goal of this study was to test the hypothesis that different interpersonal communication styles (ICSs) are differently associated with personal and professional growth. The results provide evidence consistent with the hypothesis: different ICSs are differently associated with personal and professional growth. 

According to the participants’ reports, most were able to grow professionally and personally when directed by others and when respected individuals used the controlling style in times of crisis. By contrast, fewer respondents strongly agreed that they grew both professionally and personally when encouraged to take action by their superiors or when placed in a challenging situation by a superior’s request. Sethi and Seth [22] note that the controlling style is used to “direct others and gain their compliance” and can be “effective when used on occasion by respected individuals, particularly in times of crisis” (p. 38). The controlling style is a one-way communication approach, used to direct people. Minimal or no feedback is required when using the controlling style, and individuals employing this approach use power and manipulation to underpin their message [7] (p. 539). Although the controlling style can be effective, it is also a style of communication that can intimidate the receiver, raise communication barriers, and alienate others in normal situations [7] (p. 539). However, this style can be effective in times of crisis or emergency. 

The dynamic style of interpersonal communication is an “energetic approach that uses inspiring requests to spur employees into action” [23] (p. 366). A few participants reported that they grew professionally and personally when they were encouraged to take action, or when they were placed in a challenging situation by a superior’s request. A dynamic style is a two-way form of communication that permits several actions between the sender and receiver, such as the use of inspiration to motivate action [7]. The dynamic style can be effective in times of crisis, but can be ineffective when the receiver has inadequate knowledge and experience. This may explain why participants indicated that they were unlikely to grow professionally and personally when this style was employed [23].

The withdrawal style of interpersonal communication also displayed a relatively low mean for growth, compared to the other styles. Thus, fewer respondents agreed that they grew professionally when a manager restricted their influence in discussion, or when the manager was unwilling to participate in a discussion. Typically, when employing this style, the sender avoided using their influence and legitimate power while communicating, leaving the receiver to attempt to interpret the message. This indicated indifference and unwillingness to participate or forward the communication, highlighting why it is often an ineffective managerial communication style [24].

It is also important to consider the demographic characteristics of the sample when interpreting the results. More women than men participated in the study. Merchant [25] investigated gender differences in communication styles, and found that men and women perceive the role of communication differently. Whereas women employ communication “as a tool to enhance social connections and create relationships, men use language to exert dominance and achieve tangible outcomes” [25] (p. 17). Another study addressing gender differences in communication found that women used more “affiliative speech”, which included agreement and conciliation, whereas men used more “assertive speech”, which included providing information to act on [26] (p. 7). Socialization and social/contextualist theoretical frameworks may help explain gender differences in communication styles: one such framework is Gender Socialization and Identity theory, which posits that gender is learned through socialization [27]. Future research may profitably secure data from larger samples of men and women, to afford a statistically defensible investigation of gender differences in ICSs, and associations with personal and professional growth.

Consideration of other demographic characteristics of the sample also may account for some of the results, and suggest directions for future research. For instance, the largest age group participating in the study was 31–40 years old, and most of the respondents had attained a college diploma. Moreover, all the participants reported that they were currently employed, suggesting recent encounters within their organizations that would directly inform their answers. Thus, the current results may reflect most clearly the perceptions of college-educated and currently employed Saudi Arabian adults. Future research might profitably investigate whether responses vary by education level and current employment status. Furthermore, although this is the first study to investigate the associations of ICSs with personal and professional growth in a Saudi Arabian sample, we suggest that future research investigate whether these results replicate in other Middle Eastern cultures.

## 5. Conclusions

In summary, ICSs appear to be differently associated with personal and professional growth in Saudi Arabian employees. The current results suggest that the controlling managerial style better predicts employee growth than does the dynamic or withdrawal style, for example. The controlling style is used to direct others and gain compliance, and is more effective in times of crisis, thereby facilitating employee growth. The dynamic and withdrawal styles, in contrast, require employees to interpret ambiguous or minimal managerial communication, making it difficult for employees to grow professionally or personally in the absence of clear managerial guidance or direction. The dynamic and withdrawal styles may be especially ineffective when employees are not knowledgeable and experienced. Several results of the current research parallel the results of work with Western samples, suggesting cross-cultural similarities in the relationships between ICSs and personal and professional growth. Other results may be specific to non-Western or Saudi samples, and this suggests an avenue for future research investigating the possibility of cultural differences. 

## Figures and Tables

**Table 1:** Description of interpersonal communication styles (adapted from [7]).

Interpersonal Style	Type	Description
Controlling	One-way communication	Used to direct others, to gain compliance; Anticipates little or no feedback; Employs power and manipulation; Can be intimidating, and raises communication barriers.
Egalitarian	Two-way communication	Shares information, rather than directing; Encourages others to contribute their ideas; Builds trust and positive relationships.
Structuring	Task-focused	Used to establish schedules and impose organization; Monitors compliance with company rules; Informs others of goals and objectives.
Dynamic	High-energy	Uses inspiration to motivate others to take action; Most effective in times of crisis; Is not effective if receiver is less experienced.
Relinquishing	Deferential	Highly receptive to opinions of others; Allows others to discuss, but offers minimal comments; Effective when receiver has experience and knowledge to act.
Withdrawal	Lack of communication	Sender avoids using their influence and power; Indicates unwillingness to participate or communicate.

**Table 2:** Summary of the demographic attributes of the participants.

Variable	Attribute	Total
Gender [G]	Male	68
	Female	124
	N	192
Age [A]	Less than 20	9
	21–30	24
	31–40	71
	41–50	61
	51–60	17
	More than 60	10
	N	192
Level of Education [LE]	Elementary school	13
	Middle School	23
	High school	36
	Diploma	60
	Bachelor’s degree	37
	Master’s degree	14
	Doctoral degree	9
	N	192
Monthly Income [MI]	No income	13
	Less than 5000 SR	29
	5000–9999 SR	55
	10,000–14,999 SR	57
	15,000–20,000 SR	29
	More than 20,000 SR	9
	N	192
Work Status [WS]	Yes	192
	No	0
	N	192

**Table 3:** Item indexing the effect of interpersonal communication style on personal and professional growth (adapted from [7]).

Style	Codes	Items
Controlling	CS	When respected individuals use a controlling style in times of crisis, I am able to grow personally and professionally.
Egalitarian	ES	I grow personally and professionally when I express my ideas and opinions to reach a mutual understanding.
Structuring	SS	I grow personally and professionally when there are company standards and rules to be followed.
Dynamic	DS	I grow personally and professionally when I am encouraged to take action.
Relinquishing	RS	I grow personally and professionally when a manager delegates the responsibility of communication to me.
Withdrawal	WS	I grow personally and professionally when a manager restricts his influence in a discussion.

**Table 4:** Descriptive statistics for personal and professional growth by interpersonal communication style (ICS).

ICS	Average	ICS	Level
CS1	4.2343	CS2	High
ES1	4.0138	ES2	High
SS1	4.0486	SS2	High
DS1	3.8472	DS2	Moderate
RS1	4.0277	RS2	High
WS1	3.9791	WS2	Moderate

Note: See text for full labels for ICSs.

**Table 5:** ANOVA of effects of ICS on personal and professional growth.

Sources Variation	*SS*	*Df*	*MS*	*F*	*p*-Value	*F* Critical
Between	13.25557	5	2.651115	2.264777	0.04624	2.223983
Within	1060.55	906	1.170585			
Total	1073.806	911				

## Data Availability

Due to the nature of this research, the participants did not agree for their identified data to be shared publicly.
